# Predicting the impact of stuttering in the school years

**DOI:** 10.3389/fpsyg.2026.1782752

**Published:** 2026-04-30

**Authors:** Kurt Eggers, Sharon Millard

**Affiliations:** 1 Stuttering Research Group, Department of Rehabilitation Sciences, Ghent University, Ghent, Belgium; 2 Department of Speech-Language Pathology, Thomas More University College, Antwerp, Belgium; 3 Michael Palin Centre, London, United Kingdom

**Keywords:** anxiety, depression, impact, school-age, stuttering, temperament

## Abstract

**Purpose:**

The goal of the current study was to identify factors contributing to the overall impact of stuttering, as measured by the Overall Assessment of the Speaker’s Experience of Stuttering (OASES) in older children who stutter. Prior research suggests temperament and emotional functioning may influence stuttering impact, whereas findings regarding overt stuttering severity have been inconsistent.

**Method:**

Participants were 306 children who stutter (241 boys, 65 girls), aged 9;0–14;11 years old, referred to a specialist center for stuttering. Measures included the Early Adolescent Temperament Questionnaire-Revised (EATQ-R), Stuttering Severity Instrument-4 (SSI-4), Revised Children’s Anxiety and Depression Scale (RCADS), and Overall Assessment of the Speaker’s Experience of Stuttering (OASES). A multiple linear regression was conducted with OASES total score as the outcome and predictors including: SSI-4 total score, temperament dimensions (Surgency, Negative Affect, and Effortful Control), RCADS total score, age, and sex.

**Results:**

Higher Surgency predicted lower stuttering impact, whereas higher Negative Affect, greater anxiety and depression symptoms, older age, and higher overt stuttering severity predicted greater impact. Effortful Control and sex were not significant predictors. Temperament and internalizing symptoms contributed independently to stuttering impact, beyond overt severity. Predictors operated largely independently within the multivariate model, with no evidence of strong interaction effects.

**Conclusion:**

These findings add to the understanding of the factors that contribute to the impact that stuttering has on 9–14 year olds. The results indicate that the impact that stuttering has on a child can be predicted to some degree by their temperament, level of anxiety and depression, age and overt stuttering, but not sex. Future research is needed to identify/include other factors that may strengthen this predictive model, better understand how and why some children are more negatively impacted than others, and to inform clinical practice.

## Introduction

Stuttering is a multifactorial neurodevelopmental condition characterized by disruptions in the forward flow of speech that vary widely in onset, course, and lived impact across individuals (Chang et al., 2025). Although speech disfluency is the defining feature, children who stutter (CWS) frequently report broader psychosocial consequences, including reduced participation, negative self-evaluation, and emotional distress (Briley et al., 2019; Johnson et al., 2023). Evidence suggests that the likelihood of persistence and the degree of negative impact can increase with age (e.g., Guttormsen et al., 2015). Cross-sectional research consistently shows substantial heterogeneity in adverse impact, with some people who stutter reporting minimal functional limitation despite ongoing disfluency (e.g., Tichenor and Yaruss, 2019), although longitudinal evidence tracking changes in impact over time remains limited. At the same time, not all children and adolescents who stutter experience stuttering negatively, and the degree to which stuttering affects functioning, well-being, and participation varies considerably between individuals and across time. This variability is important; those who present clinically do so because they or their parents are concerned about stuttering’s current impact or fear its future consequences, while others report minimal distress or limitation despite ongoing disfluency.

A plausible reason for this heterogeneity in stuttering is the dynamic interaction between intrinsic and extrinsic influences (e.g., Smith and Weber, 2017; Starkweather and Gottwald, 1990). Intrinsic factors include temperamental tendencies (i.e., children’s characteristic patterns of emotional reactivity and self-regulation) that shape how stressors are appraised and managed, as well as cognitive styles such as repetitive negative thinking, which has been linked to greater emotional burden in young people who stutter (Eggers et al., 2009, 2010; Rothbart, 2011; Tichenor et al., 2023). Extrinsic factors encompass external support (Boyle, 2013), family, peer, and school environments (Beilby, 2014; Johnson et al., 2023; Upadhyay et al., 2025), as well as community attitudes and stigma (Johnson et al., 2024), which can either buffer or amplify stress responses (Beilby, 2014; Boyle, 2015). Together, these intrinsic and extrinsic influences can also contribute to resilience, enabling some children to adapt positively and maintain participation and well-being despite the challenges associated with stuttering (Walsh et al., 2023). The literature on stuttering has long emphasized this interactional perspective whereby biological predispositions and environmental factors jointly contribute to the onset, variability, and lived experience of stuttering (e.g., Conture and Walden, 2012; Smith and Weber, 2017; Starkweather, 2002; Walden et al., 2012).

Consistent with contemporary models of disability and functioning, we see impact as a multidimensional construct that includes affective reactions to stuttering, participation in daily communication, and broader quality-of-life domains. The Overall Assessment of the Speaker’s Experience of Stuttering (OASES) is one measure used in both clinical practice and research to evaluate these different facets (Yaruss and Quesal, 2016). Informed by the World Health Organization’s International Classification of Functioning, Disability and Health (ICF) Model (World Health Organization, 2001), OASES conceptualizes the stuttering experience across body function/structure, activities/participation, and personal/environmental factors. For school-aged populations, the OASES-S has among the strongest measurement properties for assessing the psychological impact of stuttering, and it is frequently selected as an outcome measure in studies exploring the correlates of stuttering impact (e.g., Jones et al., 2021).

Temperament is often referred to as the lens through which a person perceives and responds to their world. Rothbart (2011) defined it as a set of constitutionally based differences in reactivity and self-regulation that are biologically rooted yet shaped by maturation and experience. While different frameworks exist, Rothbart’s model highlights three broad dimensions frequently examined in stuttering research: Surgency (approach-related traits such as high-intensity pleasure, and low fear/shyness), Negative Affect (propensity toward frustration, sadness, anger), and Effortful Control (attentional control, planning, and the capacity to regulate prepotent responses; Ellis, 2002; Ellis et al., 2004). These dimensions influence the frequency and intensity of emotions and the behavioral strategies used to manage those emotions (Rothbart and Derryberry, 1981; Heselmans and Eggers, 2025). In school-aged CWS, prior work has linked temperament to both overt stuttering characteristics and self-reported impact. For example, higher Surgency and lower Negative Affect have been associated with lower OASES scores – i.e., less adverse impact – suggesting that more extraverted, less fearful/shy children experience a lower overall impact of their stuttering, whereas children with greater irritability and frustration experience a higher overall impact of their stuttering (Eggers et al., 2021). The literature on Effortful Control is mixed, with some age-dependent or sample-dependent differences reported, while others finding no role for Effortful Control in school-aged children (Eggers et al., 2022). In a study that included children and adolescents aged 10–17 years, emotional suppression (an emotion-regulation strategy) was associated with higher OASES scores, while cognitive reappraisal was not a significant predictor (Tichenor et al., 2022). The relationship between these strategies and stuttering impact differed between cohorts of differing ages across the lifespan. The relationship between temperament, emotional regulation and stuttering impact appears therefore to be dynamic and these findings underscore the need to examine specific ages and times of life.

Temperament not only influences the frequency or intensity of an emotion, but also shapes a person’s behavioral responses to an emotion or situation. One such behavior is anticipatory and avoidant behavior. In children and adolescents who stutter (9–17 years), higher levels of shyness have been associated with more avoidant responses when anticipating moments of stuttering (Rodgers and Jackson, 2021). Although avoidance of feared speaking situations is understandable, research in related areas shows that reliance on avoidance as a primary coping strategy can restrict participation and undermine well-being. For example, Iverach et al. (2017) demonstrated that anxiety, speech dissatisfaction, and negative self-evaluations are linked to greater avoidance among individuals who stutter, and Levy Kardash et al. (2025) reported similar associations between shyness, avoidance, and reduced participation in typically developing children. Taken together, these findings suggests that temperament may shape how children feel about stuttering and how they cope with it, which, in may turn influence the overall impact stuttering has on daily functioning.

Robust evidence indicates that anxiety, particularly social anxiety, is more prevalent among CWS than among their non-stuttering peers (Bernard et al., 2022). In an Australian study (75 CWS versus 150 CWNS), 24% of CWS met diagnostic criteria for Social Anxiety Disorder compared with only 5% of controls (Iverach et al., 2017). Those with elevated social anxiety have been shown to have higher levels of bias toward threat stimuli, such as faces showing negative affect, than children and adolescents who stutter who do not have clinically significant levels of social anxiety (McAllister et al., 2015). Beyond social anxiety, in a clinical cohort of children and adolescents who stutter between the ages of 8–18, a greater proportion than would be expected scored above the scores indicating clinical significance for ‘panic’ and ‘generalized anxiety’. In the younger cohort (those aged 8–12) there were higher levels of ‘separation anxiety’ than would be expected according to population prevalence rates (McAllister et al., 2015). These findings were supported by the current authors in an investigation of anxiety in a clinical cohort of 9–14 year olds referred to a specialist center for stuttering in the UK (Eggers et al., 2022). The results showed that not only were this group more likely to experience clinically significant levels of anxiety than might be expected in the general population, but that the level of anxiety was associated with temperament: higher Negative Affect and lower Surgency/Effortful Control were linked to higher levels of anxiety and depression (Eggers et al., 2022). These observations support a plausible temperament-emotion-impact pathway, in which temperament predispositions contribute to emotional difficulties that, in turn, shape stuttering’s lived impact.

It is reasonable to anticipate that children with elevated levels of anxiety and depression may also be those who experience stuttering more negatively, through increased avoidance, diminished participation, or more negative self-appraisal, even if associations with disfluency frequency or overt severity are inconsistent across studies (e.g., Manning and Beck, 2013; Mulcahy et al., 2008). A related developmental construct, behavioral inhibition (BI) (i.e., the chronic tendency to respond to novel persons, places, and situations with wariness and avoidance) has been identified as a principal predictor of Social Anxiety Disorder, with longitudinal evidence suggesting that more than 40% of behaviorally inhibited children go on to develop SAD (Clauss and Blackford, 2012). BI overlaps conceptually with aspects of low Surgency/high fear/shyness, further supporting the role of a temperament-linked risk pathway.

The relationship between overt stuttering severity and temperament or emotional variables has been mixed. In a cohort spanning 2.5–12.5 years, reduced regulatory skills (i.e., lower Effortful Control) have been associated with increased stuttering severity as rated by parents and clinicians (Kraft et al., 2019). In contrast, studies of older children and adolescents have not consistently found straightforward links between overt severity and temperament or anxiety/depression (Eggers et al., 2021, 2022). Differences in sample characteristics, rating methods, age ranges, and cultural contexts likely contribute to these discrepancies.

Although age often represents cumulative social and developmental influences, its association with stuttering impact is not consistent across research. Some work suggests age-related differences in emotion-regulation strategies and their links to OASES scores (e.g., Tichenor et al., 2022), while other analyses have not found age to be a strong predictor (e.g., Blumgart et al., 2012; de Sonneville, 2015). This paper focuses on late childhood and early adolescence (approximately 9–14 years) as this is a developmental window in which peer evaluation and school-based communication demands intensify (Somerville, 2013). For some children, this period coincides with heightened awareness of disfluency and increased sensitivity to how others respond to it (Parker et al., 2015). Conversely, it can also be a time when coping strategies and supportive environments begin to consolidate (Berger et al., 2026). To maintain clarity and readability throughout this manuscript, we will use the term older children to refer to participants in this 9–14 year age range.

From a clinical perspective, a better understanding of why some children experience stuttering more negatively than others can inform therapeutic practice at several levels. First, identifying children at greater risk of adverse impact can help prioritize limited resources. Second, understanding how different factors contribute to the impact, and may interact with one another, can guide therapy focus and tailor intervention targets. In this context, insight in a child’s temperament can help anticipate a child’s coping style and tailor intervention accordingly. Third, an integrated view can facilitate shared decision-making with families, calibrating expectations and jointly deciding on treatment goals that address the child’s unique profile of strengths and needs. For example, knowing a child’s temperament can guide discussions with families about likely therapy needs: children low in Effortful Control may require more support for emotional regulation, whereas children high in Negative Affect may need a slower, graduated approach to challenging situations (e.g., Kristal, 2005). Such information helps clinicians and families set realistic expectations and select goals that align with the child’s regulatory and emotional profile.

The literature on stuttering impact has largely relied on pairwise associations, leaving gaps in understanding how multiple factors interact. In the 9–14 year age range, it remains unclear whether temperament dimensions (Surgency, Negative Affect, Effortful Control), anxiety and depression, overt stuttering severity, and demographics (age, sex), when considered together, predict the overall impact of stuttering. A regression-based approach can quantify their unique and combined contributions, test whether psychosocial factors outweigh overt stuttering severity, and identify risk profiles in clinical samples. Building on prior work with similar cohorts (Eggers et al., 2021, 2022), this study examines these variables simultaneously in older children referred to specialist services. By moving beyond bivariate analyses, we aim to clarify which factors most strongly predict impact and whether severity adds explanatory value once temperament and emotional variables are included.

### Objectives and hypotheses

The present study builds on earlier publications from our group with older children aged 9–14 years (Eggers et al., 2021, 2022), referred to specialist speech and language therapy services, by moving from bivariate correlations to a multivariate regression and interaction-based examination of combined and conditional effects. It aims to identify the unique and combined contributions of temperament, anxiety and depression, overt stuttering severity, age, and sex to overall impact of stuttering. Previous research suggests that temperament, as measured by the Early Adolescent Temperament Questionnaire-revised (EATQ-R) and emotional aspects as measured by the Revised Children’s Anxiety and Depression Scale (RCADS) may play a role in how stuttering is experienced, whereas findings regarding overt stuttering severity, as measured by the Stuttering Severity Instrument-4 (SSI-4) have been inconsistent. Eggers et al. (2021) reported that higher Surgency and Effortful Control, and lower Negative Affect, were associated with reduced impact, while stuttering severity showed no clear relationship. In addition, Eggers et al. (2022) found links between temperament and anxiety and depression, but not between stuttering severity and anxiety and depression. Sex was also included as a factor of interest as Samson et al. (2021) found that teenage girls were more vulnerable to the negative impact of stuttering than boys.

To extend these findings, current study will use a multiple linear regression analysis with overall stuttering impact (OASES) as the outcome variable and the following predictor variables: stuttering severity (SSI-4 total score), temperament dimensions (EATQ-R: Surgency, Negative Affect, Effortful Control), anxiety and depression (RCADS total score), age, and sex. Although our conceptual model presents temperament, internalizing symptoms, and stuttering severity as predictors of functional impact, these relationships are likely dynamic and bidirectional. Temperament and emotional symptoms may shape how children experience stuttering, but the challenges captured by the OASES may likewise reinforce emotional reactivity or anxiety over time. Given our cross-sectional design, the present study therefore focuses on one plausible direction of association while acknowledging the potential for reciprocal influences.

We hypothesize that (1) higher Surgency and Effortful Control and lower Negative Affect will predict lower overall impact as previous findings indicated that adaptive temperament profiles are associated with reduced psychosocial burden (Eggers et al., 2021); (2) higher RCADS scores will predict higher overall impact given the established link between emotional difficulties and negative self-perceptions; (3) SSI-4 severity may or may not strongly predict impact. Although intuitively greater overt stuttering severity might increase impact, prior research (e.g., Eggers et al., 2021) did not confirm this association; (4) older age will predict higher impact, reflecting cumulative psychosocial challenges over time; (5) sex effects are examined without a directional expectation. Although these hypotheses reflect expected directional associations at the predictor level, the factors under study are likely to operate jointly rather than independently. Therefore, in addition to testing their unique contributions within a multivariate framework, we also examine selected interaction terms to explore whether the effect of one predictor may depend on the level of another. This allows us to consider potential combined or conditional effects consistent with the more complex patterns suggested in the introduction.

## Methods

### Participants

Participants were 306 English-speaking children who were aged between 9; 0 and 14; 11 years (*M* = 11;6, *SD* = 1;7); 243 (79.4%) were monolingual English-speaking, 63 (20.6%) spoke more than one language. The male (*n* = 241; 78.8%) to female (*n* = 65; 21.2%) ratio was 3.7:1. They were all referred to a specialist center for children who stutter (CWS). This cohort is an extension of the previous cohorts reported in Eggers et al. (2021, 2022). Of the 306 participants, at least 174 were newly recruited; the remainder likely overlap with the 2021 and 2022 cohorts, although the exact overlap cannot be determined because not all required variables were available in those earlier datasets. As part of the standard clinical evaluation, each child completed a comprehensive assessment battery for an evaluation of their stuttering and associated influencing factors. Stuttering status was established through child self-identification, parental report, and confirmation by a speech and language therapist with expertise in stuttering. Overt stuttering severity was measured by the Stuttering Severity Instrument (SSI-4; Riley, 2009) using a speech sample of a minimum of 300 syllables during both reading and conversation activities. Sound, syllable and monosyllabic word repetitions, prolongations and blocks were included as stuttered events (Conture, 2001). The average percentage of stuttered syllables was 9.76 (*SD* = 11.39). Forty-seven participants were classified on the SSI-4 as very mild, 93 as mild, 66 as moderate, 60 as severe, and 33 very severe. The average time since stuttering onset was 6.4 years (*SD* = 2.97), with only a minority (3%) having stuttered less than a year. Most participants (*n* = 222; 72.5%) had attended stuttering therapy before attending for an assessment at this center.

Because the sample comprised a clinical population, no exclusion criteria were applied. Table 1 provides an overview of the co-existing conditions. Additional conditions are reported only if a formal diagnosis was made by professional qualified to do so. The most frequently reported additional diagnosis was autism^1^ (*n* = 26), followed by attention deficit and hyperactivity disorder (ADHD) (*n* = 10). Fourteen had two additional diagnoses and two children had three or more. There were 20 children who showed signs of other conditions (e.g., autism, ADHD, dyslexia), but were awaiting referral, assessment or diagnosis at the time the data were collected.

**Table 1:** Number and proportion of the participants with co-existing conditions.

Co-existing condition	Number of cases	Proportion of participants
None reported	208	67.8%
Autism	26	8.8%
Attention Deficit Hyperactivity Disorder	10	3.3%
Anxiety	6	2%
Dyspraxia	4	1.3%
Global Developmental Delay	4	1.6%
Learning Difficulties	7	2.35%
Developmental Language Disorder	2	0.7%
Other (e.g., hearing difficulties, asthma, eczema, kidney problems)	35	11.43%

All data were collected at the first visit to the center. In accordance with Health Research Authority guidance, this study does not require formal ethical approval, as it involves a secondary analysis of an anonymized clinical dataset. Nevertheless, to ensure transparency, all clients attending this center are informed about the potential use of their anonymized clinical data for service evaluation and research into stammering. Clients are asked to provide explicit consent for their data to be included in such analyses, with the option to decline.

### Measures

#### Early adolescent temperament questionnaire–revised

Temperament was measured using the Early Adolescent Temperament Questionnaire–Revised (EATQ-R; Ellis and Rothbart, 2001), a self-report questionnaire for adolescents aged 9–15 years that is based on Rothbart’s temperament framework. The instrument comprises 12 temperament scales that cluster into four higher-order factors: Surgency, Negative Affect, Effortful Control, and Affiliativeness (see Table 2). The EATQ-R consists of 65 items rated on a 5-point Likert scale ranging from 1 (almost never true) to 5 (almost always true). Previous research reports an average internal consistency of 0.73 for the total set of scales. Reliability coefficients exceed 0.80 for the Shyness and Aggression scales, fall between 0.60 and 0.70 for Activation Control, Affiliation, Frustration, High Intensity Pleasure, Perceptual Sensitivity, and Pleasure Sensitivity, and range from 0.65 to 0.70 for the remaining scales (Ellis and Rothbart, 2001).

**Table 2:** Definitions and sample items for the early adolescent temperament questionnaire (EATQ-R) (Ellis, 2002).

Scale	Definition and sample item
Surgency	
1. High intensity pleasure	The pleasure derived from activities involving high intensity or novelty. *Sample item: I would not be afraid to try something like mountain climbing.*
2. Fear (reverse score)	Unpleasant affect related to anticipation of distress. *Sample item*: *I worry about getting into trouble.*
3. Shyness (reverse score)	Behavioral inhibition to novelty and challenge, especially social. *Sample item: I am shy about meeting new people.*
Negative affect	
4. Frustration	Negative affect related to interruption of ongoing tasks or goal blocking. *Sample item*: *I get irritated when I have to stop doing something I’m enjoying.*
5. Depressive mood	Unpleasant affect and lowered mood, loss of enjoyment and interest in activities. *Sample item*: *My friends seem to enjoy themselves more than I do.*
6. Aggression	Hostile and aggressive actions, including person- and object-directed physical violence, direct/indirect verbal aggression, and hostile reactivity. *Sample item*: *I pick on people for no real reason.*
Effortful control	
7. Activation control	The capacity to perform an action when there is a strong tendency to avoid it. *Sample item*: *If I have a hard assignment to do, I get started right away.*
8. Attention	The capacity to focus attention as well as to shift attention when desired. *Sample item*: *I pay close attention when somebody tells me how to do something.*
9. Inhibitory control	The capacity to plan, and to suppress inappropriate responses. *Sample item*: *It’s easy for me to keep a secret.*
Affiliativeness	
10. Affiliation	The desire for warmth and closeness with others, independent of shyness or extraversion. *Sample item*: *It is important to me to have close relationships with other people.*
11. Perceptual sensitivity	Detection or perceptual awareness of slight, low-intensity stimulation in the environment. *Sample item*: *I tend to notice little changes that other people do not notice.*
12. Pleasure sensitivity	Amount of pleasure related to activities or stimuli involving low intensity, rate, complexity, novelty, and incongruity. *Sample item: I like the crunching sound of autumn leaves.*

#### Stuttering severity instrument-4

Overt stuttering severity was assessed with the SSI-4 (Riley, 2009). This standardized measure evaluates stuttering severity across multiple speech dimensions, including frequency, duration, and physical concomitants. Frequency is calculated as the percentage of syllables stuttered and subsequently converted to scale scores ranging from 4 to 18. Duration is operationalized as the mean length of the three longest stuttering events, measured to the nearest tenth of a second and likewise transformed into scale scores between 2 and 18. Physical concomitants – specifically distracting sounds, facial grimaces, head movements, and movements of the extremities – are rated on a 6-point scale from 0 (absent) to 5 (severe and painful in appearance) and converted to scale scores ranging from 0 to 20. The overall SSI-4 score is obtained by summing the individual scale scores; these scores can be translated into a severity classification spanning from very mild to very severe. For assessment purposes, speech samples obtained during both reading tasks and conversational interaction with a clinician were video recorded and subsequently analyzed.

#### Revised children’s anxiety and depression scale

Symptoms of anxiety were evaluated with the Revised Children’s Anxiety and Depression Scale (RCADS; Chorpita et al., 2000), a standardized self-administered instrument for children and adolescents between 8 and 18 years of age. The questionnaire contains 47 items and yields scores on six empirically derived scales. Five scales assess anxiety-related symptom domains – Separation Anxiety Disorder, Social Phobia, Generalized Anxiety Disorder, Panic Disorder, and Obsessive Compulsive Disorder – whereas depressive symptomatology is captured by the Major Depressive Disorder scale (see Table 3). The scale structure and item content were informed by DSM-IV diagnostic criteria for anxiety and depressive disorders. Respondents indicate symptom frequency using a 4-point response format ranging from 0 (never) to 3 (always). Reliability estimates reported in the literature indicate satisfactory to excellent internal consistency, with coefficients ranging from 0.78 for Social Anxiety Disorder to 0.88 for Panic Disorder (Chorpita et al., 2005). The RCADS has been widely adopted in both clinical and research settings, and its psychometric properties have been extensively evaluated (Ebesutani et al., 2011), including successful translation and validation in multiple languages (e.g., Gormez et al., 2017; Kösters et al., 2015). The total anxiety and depression score was computed by summing responses across all RCADS items, yielding a composite index of overall level of internalizing symptoms.

**Table 3:** Definitions and sample items of the revised children’s anxiety and depression scale (RCADS) (Chorpita et al., 2000).

Scale	Definition and sample item
Separation Anxiety Disorder	Anxiety disorder occurring in childhood or adolescence that is characterized by developmentally inappropriate, persistent, and excessive anxiety about separation from the home or from major attachment figures. *Sample item: I would feel afraid of being on my own at home.*
Generalized Anxiety Disorder	Difficult to control, long-lasting excessive anxiety and worry about a range of concerns (e.g., world events, finances, health, appearance, activities of family members and friends, work, school) accompanied by such symptoms as restlessness, fatigue, impaired concentration, irritability, muscle tension, and disturbed sleep. *Sample item: I worry that something bad will happen to me.*
Panic Disorder	Anxiety disorder characterized by recurrent, unexpected panic attacks that are associated with (a) persistent concern about having another attack, (b) worry about the possible consequences of the attacks, (c) significant change in behavior related to the attacks (e.g., avoiding situations, engaging in safety behavior, not going out alone), or (d) a combination of any or all of these. *Sample item: When I have a problem, my heart beats really fast.*
Social Phobia	Anxiety disorder characterized by recurrent, unexpected panic attacks that are associated with (a) persistent concern about having another attack, (b) worry about the possible consequences of the attacks, (c) significant change in behavior related to the attacks (e.g., avoiding situations, engaging in safety behavior, not going out alone), or (d) a combination of any or all of these. *Sample item: I worry what other people think of me.*
Obsessive Compulsive Disorder	Disorder characterized by recurrent intrusive thoughts (obsessions) that prompt the performance of neutralizing rituals (compulsions). *Sample items: I cannot seem to get bad or silly thoughts out of my head.*
Major Depressive Disorder	Mood disorder characterized by persistent sadness and other symptoms of a major depressive episode but without accompanying episodes of mania or hypomania or mixed episodes of depressive and manic or hypomanic symptoms. *Sample item: I feel worthless.*

#### Overall assessment of the speaker’s experience of stuttering

The impact of stuttering on everyday functioning was assessed using the Overall Assessment of the Speaker’s Experience of Stuttering (OASES; Yaruss and Quesal, 2016), a self-report measure that captures stuttering-related experiences from the perspective of the individual who stutters. The instrument is organized into four sections: General Information, which addresses the child’s perceived amount of stuttering, knowledge about stuttering, and self-concept as a person who stutters; Reactions to Stuttering, encompassing emotional, physiological, and cognitive responses associated with stuttering; Communication in Daily Situations, which evaluates perceived difficulty in speaking and participating across everyday communicative contexts; and Quality of Life, reflecting the extent to which stuttering interferes with and negatively affects the child’s life. Age-appropriate versions of the OASES were administered: the 60-item school-age version (OASES-S) for participants aged 9–12 years and the 80-item teenage version (OASES-T) for those aged 13–15 years. Items are rated using a 5-point Likert-type scale ranging from 1 (not at all) to 5 (completely). Reported internal consistency coefficients across the four sections range from 0.67 to 0.94 for the OASES-S and from 0.88 to 0.98 for the OASES-T (Yaruss and Quesal, 2008). Higher overall OASES scores indicate a greater negative impact of stuttering on the individual’s functioning and well-being.

### Data analyses

To examine predictors of functional stuttering impact, a multiple linear regression was conducted with OASES as the dependent variable and SSI-4, EATQ-R factors (i.e., Surgency, Negative Affect, and Effortful Control), RCADS, age, and sex as independent variables. Predictor selection was guided by both theoretical considerations and prior empirical findings. In earlier work, the EATQ-R factors Surgency, Negative Affect, and Effortful Control were significantly correlated with RCADS scores, whereas Affiliativeness was not (Eggers et al., 2022). Surgency and Negative Affect showed moderate relationships with OASES scores while no meaningful associations were observed between EATQ-R factors and SSI-4 (Eggers et al., 2021). Accordingly, the regression model included SSI-4, RCADS, three EATQ-R factors, age, and sex, allowing assessment of their unique and combined contributions to the functional impact of stuttering. Affiliativeness was excluded from the model, as prior analyses indicated it was unlikely to provide additional explanatory value. Co-existing diagnostic conditions were not incorporated into the primary analyses because the diagnostic categories available in the dataset were heterogeneous, inconsistently reported, and not aligned with the *a priori* theoretical model, which focused on temperament, internalizing symptoms, and stuttering severity. Many characteristics linked to conditions such as autism and ADHD are already captured dimensionally through the EATQ-R and RCADS, so including diagnostic labels would risk over-adjustment. These diagnoses were therefore documented descriptively but not used analytically.

In addition to the primary main-effects model, we conducted a series of exploratory but theoretically motivated interaction analyses to evaluate whether relationships between predictors and functional impact differed as a function of temperament, internalizing symptoms, stuttering severity, or age. Each interaction term (RCADS × temperament dimensions, SSI-4 × temperament dimensions, RCADS × age, SSI-4 × age) was entered into the regression model separately to preserve model stability given sample size considerations. This analytic extension allowed us to examine whether predictors operated ‘jointly’ not only through their simultaneous inclusion in the multivariate framework, but also through potential effect-modification patterns.

There were 42 (13.7%) missing values for one variable (RCADS). Missingness was mainly due to routine clinical constraints (forgetfulness), with a smaller subset due to children being unable to complete the questionnaire (e.g., tiredness, language complexity).

Missing values were treated in two ways (Rubin, 1987). First, we imputed the missing values 50 times, analyzed each imputed dataset separately and pooled the results, all using the mice R package (van Buuren and Groothuis-Oudshoorn, 2011). To assess whether results were robust to the method of handling missing data, in a second analysis, we conducted a sensitivity analysis using complete-case analysis, ignoring the missingness (essentially assuming missingness completely at random, MCAR). The reasons for reported missingness suggest missingness at random (MAR) is more plausible than MCAR, favoring multiple imputation analysis as primary, as this generally provides less biased and more efficient estimates (White et al., 2011).

Residual (linear model) and imputation diagnostics were checked, and multicollinearity was assessed for both the complete case and imputed models, with no evidence of assumption violations, multicollinearity, or convergence issues. All statistical analyses were performed using the SPSS statistical software package version 25 (IBM Corp., 2017).

## Results

The regression model was statistically significant (*p* < 0.001) and explained a substantial proportion of variance in the speaker’s overall impact of stuttering, with predictors collectively accounting for 39.1% of the variance (*R^2^* = 0.39; adjusted *R^2^* = 0.37). The adjusted *R^2^* value suggests that the model retained explanatory power without overfitting, reinforcing the robustness of these associations. In line with STROBE recommendations (von Elm et al., 2007), both the primary multiple imputation analysis and sensitivity analysis (i.e., complete case analysis) results are reported for transparency (see Table 4).

**Table 4:** Multiple linear regression results predicting OASES combined scores (*N* = 264).

Predictor	Multiple imputation analysis			Complete case analysis		
	*β*	95% CI	*p*	*β*	95% CI	*p*
(Intercept)	1.64	0.82, 2.47	<0.001	1.60	0.71, 2.48	<0.001
SSI-4	0.01	0.01, 0.02	<0.001	0.01	0.00, 0,01	<0.001
EATQ-R: Surgency	−0.25	−0.36, −0.15	<0.001	−0.23	−0.35, −0.12	<0.001
EATQ-R: Negative Affect	0.19	0.05, 0.33	0.009	0.18	0.02, 0.33	0.023
EATQ-R: Effortful Control	−0.05	−0.17, 0.07	0.4	−0.05	−0.18, 0.07	0.405
RCADS	0.01	0.01, 0.02	<0.001	0.01	0.01, 0.02	<0.001
Age (in months)	0.01	0.00, 0.01	0.005	0.01	0.00, 0.01	0.007
Sex: female	0.11	−0.02, 0.24	0.10	0.12	−0.02, −0.26	0.087

CI, confidence interval; SSI-4, stuttering severity instrument – 4; EATQ-R, Early Adolescent Temperament Questionnaire-revised; RCADS, Revised Children’s Anxiety and Depression Scale.

### Primary multiple imputation analysis results

In the imputed analyses (*m* = 50), SSI-4, Surgency, Negative Affect, RCADS and age were independently associated with OASES (SSI-4 *β* = 0.010, 95% *CI* 0.005, 0.015; Surgency *β* = −0.252, 95% *CI* −0.359, −0.145; Negative Affect *β* = 0.186, 95% *CI* 0.046, 0.327; RCADS *β* = 0.014, 95% *CI* 0.008, 0.021; Age *β* = 0.014, 95% *CI* 0.001, 0.015). Effortful Control (*β* = −0.053), and sex (*β* = 0.107) showed no associations with OASES. Full results are shown in Table 4.

### Sensitivity complete case analysis results

The complete-case analysis (*n* = 264) yielded estimates that were similar in direction and magnitude to multiple imputation (e.g., SSI-4 *β* = 0.01 vs. 0.01; Surgency *β* = −0.23 vs. −0.25; Effortful Control *β* = −0.05 vs. −0.05) documenting robustness of the findings. The conclusions were unchanged.

Model fit indices (*R*^2^ and adjusted *R*^2^) are reported for the complete case analysis only, as these statistics are not routinely pooled across imputations (van Buuren, 2018).

**Figure 1:**
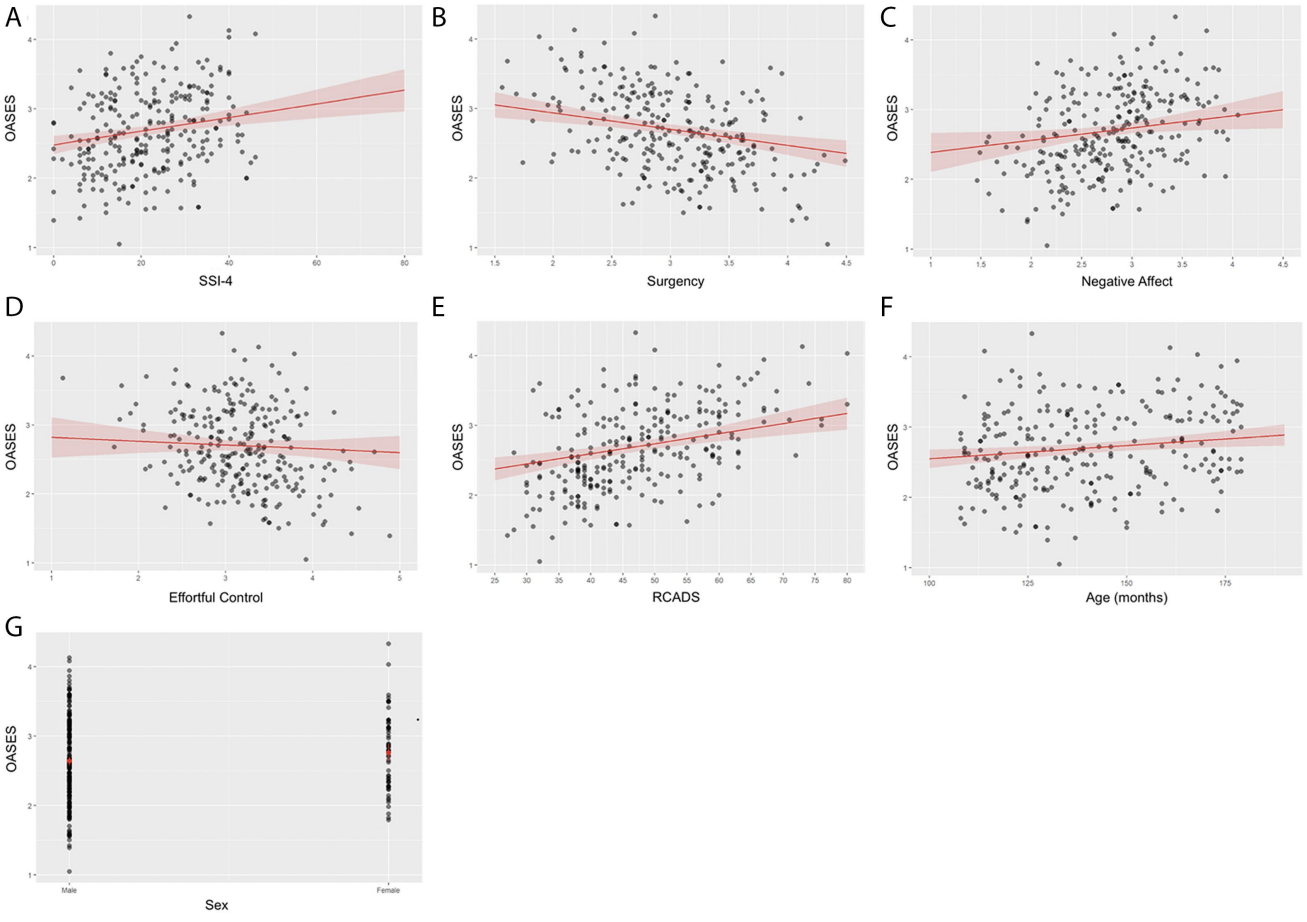
Predicted OASES scores from the multiple regression model across SSI-4, EATQ-R, RCADS, age, and sex values. Panels show the bivariate relationship between OASES scores and **(A)** SSI-4, **(B)** surgency, **(C)** negative Affect, **(D)** Effortful Control, **(E)** RCADS, **(F)** age, and **(G)** sex. Shaded areas represent 95% confidence intervals around the predicted regression line.

Figure 1 shows the predicted OASES scores across the range of SSI-4, RCADS, EATQ-R factors, age, and sex. Higher SSI-4, Negative Affect, and RCADS scores, as well as age were associated with greater negative experience of stuttering. In contrast, higher Surgency scores, were associated with lower negative experience.

**Figure 2:**
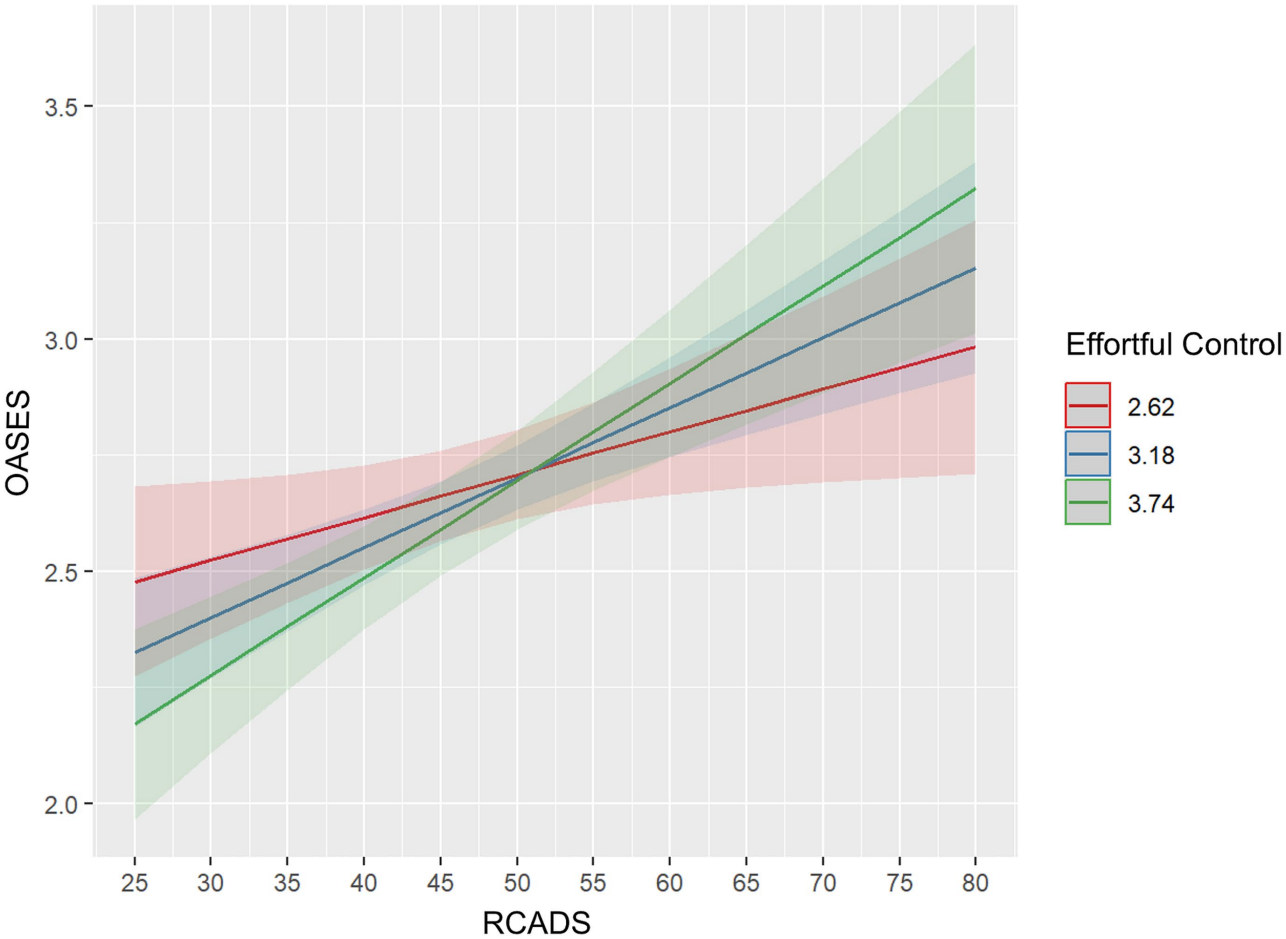
Predicted OASES scores across RCADS levels by effortful control.

Exploratory interaction models indicated that, with one exception, none of the tested interaction terms contributed significantly to the prediction of functional impact (*p* > 0.05) (see Table 5). In contrast, the interaction between RCADS and Effortful Control reached significance, indicating that higher internalizing symptoms were associated with a greater functional impact of stuttering particularly among children with lower effortful control (see Figure 2). No other interaction term improved model fit beyond the main-effects specification.

**Table 5:** Interaction effects predicting OASES combined scores (*N* = 264).

Interaction term	*β*	95% CI	*p*
RCADS × Surgency	−0.001	−0.009, 0.007	0.90
RCADS × Negative Affect	−0.006	−0.014, 0.003	0.20
RCADS × Effortful Control	0.011	0.002, 0.019	0.0012
SSI-4 × Surgency	0.002	−0.006, 0.011	0.60
SSI-4 × Negative Affect	0.001	−0.008, 0.009	0.80
SSI-4 × Effortful Control	0.001	−0.008, 0.009	0.80
Age × SSI-4	0.000	0.000, 0.000	0.80
Age × RCADS	0.000	0.000, 0.000	0.80

CI, confidence interval; SSI-4, stuttering severity instrument – 4; EATQ-R, Early Adolescent Temperament Questionnaire-revised; RCADS, Revised Children’s Anxiety and Depression Scale. Only interaction terms are displayed; all models included the same covariates as in Table 4 (surgency, negative affect, effortful control, RCADS, SSI-4, age, sex).

## Discussion

The aim of this study was to examine whether temperament (Surgency, Negative Affect, Effortful Control), anxiety and depression (RCADS total), overt stuttering severity (SSI-4), age, and sex jointly predict the overall impact of stuttering (OASES) in older children referred to specialist speech and language therapy services, and to determine the unique contribution of each factor once the others are taken into account. By applying multiple linear regression and robust procedures for missing-data handling, we aimed to move beyond simple bivariate correlations and toward an integrated multivariate model of risk factors for impact during the school years.

This study has one of the largest participant groups investigating the stuttering in older children. The male to female ratio is consistent with the data reported in the literature (Yairi and Ambrose, 2013) and suggests that the proportion of males and females that are seeking help with their stuttering is relative to the prevalence in the population. Almost three quarters of this group had accessed therapy previously and when considering these results it is important to remember that they had been referred for specialist advice and support. Interestingly, 3% had been stuttering for less than a year, despite the age range.

Approximately one third had received diagnoses in addition to stuttering, although it is recognized that, given the difficulties in accessing services and the necessary assessments, these are likely to be under-reported in these data. With regard to autism, almost 9% of this cohort had received a diagnosis of autism. This proportion is consistent with previous work showing similarly elevated autism rates in clinical samples of CWS; for example, Briley and Ellis (2018) reported that approximately 8% of CWS in their national dataset had an autism diagnosis. In a population study by Taylor et al. (2013), the incidence of autism in 8 year olds in the UK population was estimated at 0.0012% in boys and 0.0002% in girls, even the slightly higher prevalence rates of 0.0038 and 0.0008% respectively, fall considerably short of the levels reported in this current population. There may be other reasons that explain the apparent elevated incidences of co-existing conditions in older children who stutter, rather than a true association, but what is clear is that children seeking support from therapy services are often presenting with additional conditions which will have ramifications for their confidence and ability to communicate. These data demonstrate that therapists also need knowledge of these co-existing communicative differences, and understand the interaction between these and the impact on communication and well-being.

The regression model was significant, indicating that temperament traits (Surgency and Negative Affectivity), emotional factors such as anxiety and depression, but also overt stuttering severity and age are important contributors to the impact of stuttering on an older child’s daily life. Effortful Control and sex showed no independent association with OASES in this age group. Taken together, these results underscore previous research that impact is not reducible to overt stuttering severity (Beilby, 2014; Engelen et al., 2024), but it does have a role for this age group. Children’s temperament profiles, especially tendencies toward approach/positive engagement (i.e., Surgency) and toward irritability/frustration (i.e., Negative Affect), and their current internalizing symptoms (anxiety and depression) contribute meaningfully to how stuttering is experienced in daily life. The finding that Surgency is protective (lower impact) and Negative Affect and anxiety and depression are risk-enhancing (greater impact) is consistent with an appraisal-and-coping account in which temperamental biases influence threat evaluation, anticipatory anxiety (Thompson et al., 2014), and the selection of coping strategies such as approach vs. avoidance (Mairet et al., 2014). The positive age association suggests that cumulative psychosocial challenges over the school years may magnify impact, even within a restricted age range (e.g., Hardi et al., 2025).

By adding interaction terms, we also examined how these predictors might operate jointly. Apart from a modest RCADS × Effortful Control interaction, all interactions were non-significant, indicating that predictors generally acted as independent, additive contributors to children’s perceived impact. The single significant interaction suggests that internalizing symptoms may be more influential for children with lower regulatory capacity, although the modest size of this effect means that the main-effects model provides the most robust account. Although Effortful Control did not show a significant main effect in predicting functional impact, it did significantly moderate the association between internalizing symptoms and OASES scores, indicating that Effortful Control does not directly reduce stuttering impact but instead buffers children against the functional consequences of anxiety and depressive symptoms. This buffering role of effortful control is consistent with broader temperament research (Rothbart et al., 2003) and findings in other developmental domains (Winebrake et al., 2025) that has shown that higher effortful control supports adaptive emotion regulation and reduces the extent to which negative affect or stress translate into maladaptive outcomes.

### Surgency acts as a protective buffer, negative affect as a risk factor

Earlier correlational work indicated that higher Surgency (greater sociability, positive affect, lower fear/shyness) tends to co-occur with lower impact, while higher Negative Affect (frustration, irritability) co-occurs with higher impact in school-aged children (Eggers et al., 2021). The current multivariate analysis confirms these relationships when other relevant variables are included, strengthening the case that these temperament factors show independent association with the lived experience of stuttering. Children with high Surgency may be more likely to engage and feel more comfortable in different speaking situations (Rothbart et al., 1994), may have greater tolerance for brief social discomfort (e.g., Heselmans and Eggers, 2024), and may continue speaking even when they expect to stutter. In contrast, children high in Negative Affect may appraise their stuttering episodes and listener reactions through a more negative lens (Creasey et al., 1997), experience stronger autonomic arousal (Laurent et al., 2011), and may more often select avoidant coping strategies (e.g., situation withdrawal; Heselmans and Eggers, 2025), which may be associated with higher OASES impact scores (Bleek et al., 2012). This maps onto findings by Sanmartín et al. (2018), who identified Surgency and Negative Affect as predictors of social functioning.

The non-significant role of Effortful Control was unexpected. Although effortful regulation of attention and behavior plays a critical role in modulating stress (Bridgett et al., 2013) and in adaptive coping with environmental demands (Rothbart et al., 2007), the non-significant finding raises questions about its role. One possibility is that its effect is indirect, for example, operating through a reduction of internalizing symptoms (RCADS). This is plausible because a significant negative correlation was previously found between Effortful Control and RCADS (Eggers et al., 2021). Another explanation is that its influence is age-dependent, being more prominent earlier in development. Finally, its effect may be reduced once temperament reactivity and anxiety/depression are accounted for. This interpretation is consistent with prior work indicating that emotion-regulation strategies (e.g., expressive suppression) relate to higher impact, but that more fine-grained regulatory processes can vary by age group (e.g., Tichenor et al., 2022). Consequently, Effortful Control may matter more in late childhood than in early adolescence (Sanchis-Sanchis et al., 2020; Theurel and Gentaz, 2018), explaining why in our participant group, spanning 9–14 years, such age-specific effects may average out.

### Anxiety and depression predict greater stuttering impact

The RCADS total score emerged as an independent predictor of impact, consistent with models in which internalizing symptoms are associated with more negative self-evaluations, attentional bias toward threat, and avoidance of speaking contexts (core OASES components). This is consistent with findings of increased threat-related attentional bias in school-age CWS (Eichorn and Campanelli, 2025; McAllister et al., 2015). Clinically, this finding argues for routine screening of anxiety and depression in referrals for stuttering, and the importance of incorporating cognitive behavior therapy-based strategies in stuttering therapy. Examples of such strategies are psychoeducation about stuttering, cognitive restructuring (e.g., challenging negative stuttering-related thoughts), gradual exposure to speaking situations, and behavioral activation (see, e.g., Block et al., 2025; Kelman et al., 2023). Our prior work showed links between temperament profiles and RCADS (Eggers et al., 2021), implying that children high in Negative Affect and low in Surgency/Effortful Control may be double-exposed: temperament is associated with higher anxiety/depression, which is in turn associated with greater impact. The current regression suggests that both levels (i.e., temperament and anxiety and depression symptoms) remain independently associated when modeled together, but any directional pathway between them, for example, whether temperament increases vulnerability to internalizing symptoms (e.g., Caspi et al., 1995; Rothbart and Bates, 2006) that contribute to higher impact, cannot be inferred from this cross-sectional design and would require longitudinal follow-up. Although RCADS significantly predicted impact, its standardized effect was small, comparable in magnitude to the small effect of SSI-4.

### Overt stuttering severity and impact: related, but not determining

Contrary to several previous findings reporting weak (e.g., Chun et al., 2010; Horton et al., 2024) or absent associations (Chon, 2023; Iimura et al., 2025) between overt stuttering severity and impact, our model identified a small but reliable positive association between SSI-4 and OASES. At the same time, temperament factors (Surgency and Negative Affect) showed substantially larger associations with impact, whereas both internalizing symptoms and overt stuttering severity both showed small but statistically reliable associations, with effect sizes that were very similar in magnitude. This seems to suggest that, within the 9- to 14-year clinical referral cohort, impact is heterogeneous: for some children, it may be largely independent of overt severity and shaped instead by appraisal/coping and environmental responses; for others, the degree of overt stuttering contributes modestly to negative experience.

In our view, two mechanisms may help explain this variability. First, social-environmental factors, such as family support, peer attitudes, and school climate, likely vary widely: supportive contexts can buffer the effect of severity on impact, whereas unsupportive contexts may amplify it (Beilby, 2014; Johnson et al., 2023; Upadhyay et al., 2025). Second, developmental timing may play a role: in adolescents, heightened self-consciousness (and self-stigma) and peer evaluation may increase the salience of speech differences, so even modest increases in disfluency may translate into greater participation constraints than they would for younger children (Johnson et al., 2024; Palombo et al., 2025). These explanations remain tentative given the cross-sectional design, and our exploratory age × SSI-4 interaction did not show evidence of moderation in this sample. Longitudinal research will be needed to determine whether developmental timing or contextual factors truly shape how stuttering severity relates to impact.

### Age and sex

The observed positive age effect, even within the narrow 9– to 14-year age range, indicates that as children progress through school, the cumulative burden of stuttering-related challenges increases. Given that age was expressed in months, the effect size is small in magnitude (for every additional year, OASES increases by 0.12 points), though statistically reliable. Educational transitions, such as moving to secondary school, likely add social complexity that interacts with temperament and anxiety to elevate impact. This aligns with previous findings by Briley (2019) and Samson et al. (2022), which also document age-related effects on stuttering impact, and by Iverach et al. (2017) who reported a trend in this direction; however, based on their conservative Bonferroni-adjusted alpha (*p* < 0.006), their model only approached significance.

Sex was non-significant. This maps onto findings from Guttormsen et al.’s (2015) meta-analyses reporting no sex-differences on the impact of stuttering in 3- to 18-year-olds, albeit that most of the included studies focused on preschoolers. It differs from more recent findings by Samson et al. (2021) who found teenage girls to be more vulnerable to the negative impact of stuttering than boys. Possible explanations could be age, recruitment, and cultural differences. Sex or gender-related differences in impact may emerge later in adolescence, when peer evaluation intensifies (their participants were 13- to 17-year-olds). Recruitment differences may also matter: our clinically referred sample may have reduced variability across sexes, whereas some of their participants were recruited via a national stuttering association and not necessarily in treatment. Finally, cultural differences between the UK and Sweden may influence how sex interacts with stuttering impact.

#### Clinical implications

The findings indicate that these factors explain nearly 40% of the variance in older children’s negative experiences of stuttering. There are clearly other factors that contribute to stuttering impact, which are not routinely assessed in this clinical setting, despite adherence to the multi-dimensional assessment framework recommended by Brundage et al. (2021). Other factors that could enhance prediction of OASES scores may include stigma (Johnson et al., 2024), resilience (Walsh et al., 2023), external support (Boyle, 2013), family support, peer attitudes, and school culture (Beilby, 2014; Johnson et al., 2023; Upadhyay et al., 2025). While clinicians should avoid relying solely on these findings to predict adverse impact, the results strongly underline the importance of a comprehensive assessment process and a multifaceted therapeutic approach to effectively support older children who stutter.

Importantly, Surgency emerged as the strongest predictor in the model, with higher Surgency associated with lower impact. While temperament itself may not necessarily be a direct treatment target, several Surgency-related behaviors, such as confident approach, positive affect, and engagement in social communication, can be supported within therapy. Contemporary accounts of temperament acknowledge that these tendencies are shaped partly through experience (e.g., Gartstein et al., 2024; Rothbart, 2011); thus, clinicians may strengthen adaptive components of Surgency by encouraging gradual approach behaviors, reinforcing positive communicative experiences, and helping children build tolerance for mild social discomfort (e.g., Kristal, 2005). These strategies align closely with, for example, elements of the CARE Model of Treatment for Stuttering (Byrd et al., 2024), which emphasizes agency, communication confidence, and resilience rather than a narrow focus on fluency. The CARE model foregrounds strengths-based engagement, opportunities for meaningful communication, and experiential learning to support positive communication identities, all principles highly compatible with supporting children who show lower Surgency profiles.

It is reasonable to anticipate that addressing these key predictive factors within therapy may help reduce aspects of the overall impact of stuttering. Interventions such as cognitive behavior therapy and mindfulness (e.g., Kelman et al., 2023; Harley, 2018) specifically target attentional bias and negative automatic thinking underpinning social anxiety, as well as the ability to tolerate and moderate emotional reactivity. Given the smaller association, it is important not to over emphasize the finding that overt stuttering is associated with negative consequences and assume that a focus on reducing overt stuttering will lead to a reduction in stuttering impact. But neither should the relationship be ignored and it would seem reasonable to suggest that for some children, therapy which incorporates strategies to reduce struggle when speaking should be considered as part of a multidimensional approach, to maximize outcomes (Onslow, 2025; Vanryckeghem, 2025).

Further, the data show that many older children seeking help have additional co-diagnoses. These will impact on their ability to communicate and will perhaps alter their priorities for therapy and the aspects of therapy which they may find more or less helpful. The need for individualizing therapy and adapting methods is strongly supported.

#### Limitations and future directions

It is important to note that the associations observed here do not imply unidirectional influences. Contemporary models emphasize reciprocal processes (e.g., Feldman, 2015; Liu et al., 2024), and it is plausible that functional impact also contributes to heightened Negative Affectivity or internalizing symptoms. Our cross-sectional data cannot disentangle these dynamics; thus, we interpret the findings as conditional associations within one direction while recognizing that bidirectional pathways warrant investigation in future longitudinal work.

Some limitations are related to the characteristics of the assessment instruments and the clinical context in which the data were collected. For example, although overt stuttering severity was assessed from video-recorded samples by highly experienced stuttering therapists, the clinical nature of the data collection meant that rater identifiers were not retained, preventing calculation of inter-rater reliability. Although the OASES-S and OASES-T differ in item count and age-appropriate wording, both forms were developed from the same ICF-based framework and share identical section structure, scoring procedures, and impact ratings, functioning as parallel instruments designed to assess the same construct across ages 7–17. Because formal equivalence data at the 12–13 age boundary are limited, minor measurement differences may exist. We therefore acknowledge this as a limitation, although combining both versions is consistent with the instrument’s intended clinical and research use (Yaruss and Quesal, 2016).

The data analyzed in this study were drawn from routine clinical assessments and examined retrospectively. As is common in clinical datasets, some information was missing, which reduced the final sample size compared to the total number of children who attended the clinic. Children or parents who did not complete all required questionnaires may represent a distinct subgroup that is now excluded from the analysis. Additionally, by the fact that the sample reflects a clinical population, the external validity may be limited and does not necessarily represent the broader group of CWS. Children who attend a clinic for assessment and therapy are likely experiencing some degree of impact from their stuttering. Those for whom stuttering poses little or no problem would be unlikely to seek help. Consequently, this study probably underrepresents children with very low impact scores, and insights into the factors that contribute to that may be incomplete. Ideally, future research could include both clinical and non-clinical populations to provide a more comprehensive understanding of the factors that predict the existence of a negative experience of stuttering.

Some additional limitations should be noted. Given that the model explains approximately 40% of the variance, as discussed previously, there are additional unmeasured yet potentially influential contextual factors likely contribute. Further research is required to examine additional age ranges to determine whether patterns differ across developmental stages, incorporate models that include additional contextual factors, and employ longitudinal or implementation designs, to build our understanding of stuttering impact, how it develops and evolves over time. Future clinical effectiveness and efficacy studies should incorporate measures of variables that predict impact in order to determine whether they change with therapy and/or predict therapy outcome.

## Conclusion

In summary, this study demonstrates that temperament and internalizing symptoms are independently associated with the impact of stuttering during the school years, alongside – but not reducible to – overt stuttering severity. More specifically, higher Surgency is associated with lower impact, while higher Negative Affect, higher anxiety and depression symptoms, older age, and higher overt stuttering severity are associated with greater impact; Effortful Control and sex were not predictive in this 9- to 14-year-old cohort. Clinically, these findings support multidimensional assessment and integrated intervention that addresses overt stuttering symptoms, emotional symptoms, and coping, with attention to temperament-informed risk profiles. Surgency and Negative Affect showed the largest associations in the multivariate model, whereas age, RCADS, and SSI-4 each contributed small but statistically reliable effects. Future longitudinal work will be valuable to refine mechanisms and optimize personalized care.

## Data Availability

The raw data supporting the conclusions of this article will be made available by the authors, without undue reservation.

## Footnotes

1 The term “autism” is used in place of “autism spectrum disorder” in this manuscript. This follows usage common in autistic communities, based on the view that “autism” is less pathologizing and more identity-respecting (Archibald et al., 2024).

## References

[ref1] Archibald D. Dwyer P. Bury S. M. (2024). “Current debates on autism language preferences: an overview,” in The Palgrave Encyclopedia of Disability, eds. Bennett G. Goodall E. (Cham: Palgrave Macmillan).

[ref2] Beilby J. M. (2014). Psychosocial impact of living with a stuttering disorder: knowing is not enough. Semin. Speech Lang. 35, 132–143. doi: 10.1055/s-0034-1371756, 24782275

[ref3] Berger C. Hanish L. D. Cavell T. A. (2026). Editorial: the importance of peers: making the most of peer relationships in childhood and adolescence. Front. Dev. Psychol. 4:1768414. doi: 10.3389/fdpys.2026.1768414

[ref4] Bernard R. Hofslundsengen H. Frazier Norbury C. (2022). Anxiety and depression symptoms in children and adolescents who stutter: a systematic review and meta-analysis. J. Speech Lang. Hear. Res. 65, 624–644. doi: 10.1044/2021_JSLHR-21-00236, 35084999

[ref5] Bleek B. Reuter M. Yaruss J. S. Cook S. Faber J. Montag C. (2012). Relationships between personality characteristics of people who stutter and the impact of stuttering on everyday life. J. Fluen. Disord. 37, 325–333. doi: 10.1016/j.jfludis.2012.07.003, 23218215

[ref6] Block S. Brosh S. Craig A. Eggers K. Euler H. Georgieva D. . (2025). “Rehabilitation and prognosis of speech fluency disorders,” in Phoniatrics II. European Manual of Medicine, eds. am Zehnhoff-Dinnesen A. Sopko J. Monfrais-Pfauwadel M. C. Neumann K. (Cham: Springer).

[ref7] Blumgart E. Tran Y. Yaruss J. S. Craig A. (2012). Australian normative data for the overall assessment of the speaker’s experience of stuttering. J. Fluen. Disord. 37, 83–90. doi: 10.1016/j.jfludis.2011.12.002, 22531284

[ref8] Boyle M. P. (2013). Psychological characteristics and perceptions of stuttering of adults who stutter with and without support group experience. J. Fluen. Disord. 38, 368–381. doi: 10.1016/j.jfludis.2013.09.001, 24331244

[ref9] Boyle M. P. (2015). Relationships between psychosocial factors and quality of life for adults who stutter. Am. J. Speech-Lang. Pathol. 24, 1–10. doi: 10.1044/2014_AJSLP-14-0089, 25410098

[ref10] Bridgett D. J. Oddi K. B. Laake L. M. Murdock K. W. Bachmann M. N. (2013). Integrating and differentiating aspects of self-regulation: effortful control, executive functioning, and links to negative affectivity. Emotion 13, 47–63. doi: 10.1037/a0029536, 22906086

[ref11] Briley P. M. (2019) Relative Contribution of the Stuttering impact scores of the OASES (Doctoral dissertation, East Carolina University) East Carolina University. Available online at: http://hdl.handle.net/10342/7215 (Accessed March 24, 2026)

[ref12] Briley P. M. Ellis C. (2018). The coexistence of disabling conditions in children who stutter: evidence from the National Health Interview Survey. J. Speech Lang. Hear. Res. 61, 2895–2905. doi: 10.1044/2018_JSLHR-S-17-0378, 30458520

[ref13] Briley P. M. O’Brien K. Ellis C. (2019). Behavioral, emotional, and social well-being in children who stutter: evidence from the National Health Interview Survey. J. Dev. Phys. Disabil. 31, 39–53. doi: 10.1007/s10882-018-9625-x

[ref14] Brundage S. B. Ratner N. B. Boyle M. P. Eggers K. Everard R. Franken M. C. . (2021). Consensus guidelines for the assessments of individuals who stutter across the lifespan. Am. J. Speech Lang. Pathol. 30, 2379–2393. doi: 10.1044/2021_AJSLP-21-00107, 34516299 PMC9132036

[ref15] Byrd C. T. Coalson G. A. Conture E. G. (2024). CARE model of treatment for stuttering: theory, assumptions, and preliminary findings. Front. Psychol. 15:1488328. doi: 10.3389/fpsyg.2024.1488328, 39720681 PMC11667897

[ref16] Caspi A. Henry B. McGee R. O. Moffitt T. E. Silva P. A. (1995). Temperamental origins of child and adolescent behavior problems: from age three to age fifteen. Child Dev. 66, 55–68. doi: 10.1111/j.1467-8624.1995.tb00855.x, 7497829

[ref17] Chang S.-E. Below J. E. Chow H. M. Guenther F. H. Hampton Wray A. M. Jackson E. S. . (2025). Stuttering: our current knowledge, research opportunities, and ways to address critical gaps. Neurobiol. Lang. 6:nol_a_00162. doi: 10.1162/nol_a_00162, 40201450 PMC11977836

[ref18] Chon H. C. (2023). Correlation between overt and covert characteristics of stuttering in adults who stutter. Phonet. Speech Sci. 15, 35–43. doi: 10.13064/KSSS.2023.15.4.035

[ref19] Chorpita B. F. Moffitt C. E. Gray J. (2005). Psychometric properties of the revised child anxiety and depression scale in a clinical sample. Behav. Res. Ther. 43, 309–322. doi: 10.1016/j.brat.2004.02.004, 15680928

[ref20] Chorpita B. F. Yim L. Moffitt C. Umemoto L. A. Francis S. E. (2000). Assessment of symptoms of DSM-IV anxiety and depression in children: a revised child anxiety and depression scale. Behav. Res. Ther. 38, 835–855. doi: 10.1016/s0005-7967(99)00130-8, 10937431

[ref21] Chun R. Y. S. Mendes C. D. Yaruss J. S. Quesal R. W. (2010). The impact of stuttering on quality of life of children and adolescents. Pró-Fono Rev Atualização Científica 22, 410–419. doi: 10.1590/s0104-56872010000400035, 21271118

[ref9001] Clauss J. A. Blackford J. U. (2012). Behavioral inhibition and risk for developing social anxiety disorder: a meta-analytic study. J Am Acad Child Adolesc Psychiatry. doi: 10.1016/j.jaac.2012.08.002 PMC3611590 23021481

[ref22] Conture E. G. (2001). Stuttering: Its Nature, Diagnosis, and Treatment. Boston: Allyn & Bacon.

[ref9002] Conture E. G. Walden T. A. (2012). Dual diathesis-stressor model of stuttering. In: Filatova Y. O. (Editor). Theoretical issues of fluency disorders. Moscow: National Book Centre. 94–127.

[ref9003] IBM, Corp. (2017). IBM SPSS Statistics for Windows (Version 25.0) [Computer software]. IBM Corp.

[ref23] Creasey G. Ottlinger K. DeVico K. Murray T. Harvey A. Hesson-McInnis M. (1997). Children’s affective responses, cognitive appraisals, and coping strategies in response to the negative affect of parents and peers. J. Exp. Child Psychol. 67, 39–56. doi: 10.1006/jecp.1997.2396, 9344486

[ref24] de Sonneville C. (2015). The Impact and Treatment of Developmental Stuttering. Available online at: http://hdl.handle.net/1765/78589 (Accessed January 5, 2026)

[ref25] Ebesutani C. Chorpita B. F. Higa-McMillan C. K. Nakamura B. J. Regan J. Lynch R. E. (2011). A psychometric analysis of the revised child anxiety and depression scales—parent version in a school sample. J. Abnorm. Child Psychol. 39, 173–185. doi: 10.1007/s10802-010-9460-8, 20878460 PMC3041919

[ref26] Eggers K. De Nil L. F. van Den Bergh B. R. H. (2009). Factorial temperament structure of stuttering, voice disordered, and normal speaking children. J. Speech Lang. Hear. Res. 52, 1610–1622. doi: 10.1044/1092-4388(2009/07-0065), 19717650

[ref27] Eggers K. De Nil L. F. van Den Bergh B. R. H. (2010). Temperament dimensions of stuttering, voice disordered, and normal speaking children. J. Fluen. Disord. 35, 355–372. doi: 10.1016/j.jfludis.2010.10.004, 21130269

[ref28] Eggers K. Millard S. Kelman E. (2021). Temperament and the impact of stuttering in adolescents. J. Speech Lang. Hear. Res. 64, 417–432. doi: 10.1044/2020_JSLHR-20-00095, 33465312

[ref29] Eggers K. Millard S. Kelman E. (2022). Temperament, anxiety, and depression in school-age children who stutter. J. Commun. Disord. 97:106218. doi: 10.1016/j.jcomdis.2022.106218, 35597191

[ref30] Eichorn N. Campanelli L. (2025). Attention bias in school-age children who stutter: evidence from a dot-probe task. J. Speech Lang. Hear. Res. 68, 3155–3170. doi: 10.1044/2025_JSLHR-24-00686, 40540730 PMC12263188

[ref32] Ellis L. Rothbart M. (2001). “Revision of the early adolescent temperament questionnaire,” in Poster Presented at the 2001 Biennal Meeting of the Society for Research in Child Development.

[ref31] Ellis L. K. (2002). Individual Differences and Adolescent Psychological Development. Unpublished doctoral dissertation. Eugene, OR, USA: University of Oregon.

[ref33] Ellis L. K. Rothbart M. K. Posner M. I. (2004). Individual differences in executive attention predict self-regulation and adolescent psychosocial behaviors. Ann. N. Y. Acad. Sci. 1021, 337–340. doi: 10.1196/annals.1308.041 15251906

[ref34] Engelen M. M. Franken M.-C. J. P. Stipdonk L. W. Horton S. E. Jackson V. E. Reilly S. . (2024). The association between stuttering burden and psychosocial aspects of life in adults. J. Speech Lang. Hear. Res. 67, 1385–1399. doi: 10.1044/2024_JSLHR-23-00562 38625147

[ref35] Feldman R. (2015). Mutual influences between child emotion regulation and parent-child reciprocity support development across the first 10 years of life: implications for developmental psychopathology. Dev. Psychopathol. 27, 1007–1023. doi: 10.1017/S0954579415000656, 26439059

[ref36] Gartstein M. A. Kirchhoff C. M. Lowe M. E. (2024). “Individual differences in temperament: a developmental perspective,” in WAIMH Handbook of Infant and Early Childhood Mental Health, eds. Osofsky J. D. Fitzgerald H. E. Keren M. Puura K. (Cham: Springer).

[ref37] Gormez V. Kilincaslan A. Ebesutani C. Orengul A. C. Kaya I. Ceri V. . (2017). Psychometric properties of the parent version of the revised child anxiety and depression scale in a clinical sample of Turkish children and adolescents. Child Psychiatry Hum. Dev. 48, 922–933. doi: 10.1007/s10578-017-0716-1, 28251450

[ref38] Guttormsen L. S. Kefalianos E. Naess K. A. (2015). Communication attitudes in children who stutter: a meta-analytic review. J. Fluen. Disord. 46, 1–14. doi: 10.1016/j.jfludis.2015.08.001 26365773

[ref39] Hardi F. A. Peckins M. K. Mitchell C. McLoyd V. Brooks-Gunn J. Hyde L. W. . (2025). Childhood adversity and adolescent mental health: examining cumulative and specificity effects across contexts and developmental timing. Dev. Psychopathol. 37, 1954–1970. doi: 10.1017/S0954579424001512, 39359017 PMC11965435

[ref40] Harley J. (2018). The role of attention in therapy for children and adolescents who stutter: cognitive behavioral therapy and mindfulness-based interventions. Am. J. Speech Lang. Pathol. 27, 1139–1151. doi: 10.1044/2018_AJSLP-ODC11-17-0196, 30347059

[ref41] Heselmans I. Eggers K. (2024). Delay frustration in children who do and do not stutter: a preliminary study. J. Commun. Disord. 107:106403. doi: 10.1016/j.jcomdis.2023.106403, 38101316

[ref42] Heselmans I. Eggers K. (2025). “Do preschoolers who stutter use different emotion regulation strategies than preschoolers who do not stutter?” in Poster Presented at the Annual Convention of the American, Speech-Language and Hearing Association, (Washington D.C.).

[ref43] Horton S. Jackson V. Boyce J. Franken M. C. Siemers S. John M. S. . (2024). Self-reported stuttering severity is accurate: informing methods for large-scale data collection in stuttering. J. Speech Lang. Hear. Res. 67, 4015–4024. doi: 10.1044/2023_JSLHR-23-00081, 38052068

[ref44] Iimura D. Sakai N. Miyamoto S. (2025). An exploratory investigation of the association between severities of the adverse impact of stuttering and co-occurring disorders. Speech Lang. Hear. 28, 1–7. doi: 10.1080/2050571X.2024.2371661

[ref45] Iverach L. Lowe R. Jones M. O’Brian S. Menzies R. G. Packman A. . (2017). A speech and psychological profile of treatment-seeking adolescents who stutter. J. Fluen. Disord. 51, 24–38. doi: 10.1016/j.jfludis.2016.11.001, 28212718

[ref46] Johnson C. A. Gerwin K. L. Tichenor S. E. Boyle M. P. Walsh B. (2024). Evaluating stuttering self-stigma and its relationship to adverse impact in children and adolescents with the child stuttering self-stigma scale. J. Speech Lang. Hear. Res. 67, 2920–2934. doi: 10.1044/2024_jslhr-24-00069, 39141882 PMC11427442

[ref47] Johnson G. Onslow M. Horton S. Kefalianos E. (2023). Psychosocial features of stuttering for school-age children: a systematic review. Int. J. Lang. Commun. Disord. 58, 1829–1845. doi: 10.1111/1460-6984.12887, 37132231

[ref48] Jones M. L. Menzies R. G. Onslow M. Lowe R. O'Brian S. Packman A. (2021). Measures of psychological impacts of stuttering in young school-age children: a systematic review. J. Speech Lang. Hear. Res. 64, 1918–1928. doi: 10.1044/2021_JSLHR-20-00455, 34019770

[ref49] Kelman E. Berquez A. Caughter S. (2023). “Cognitive approaches with children who stutter and their parents,” in Clinical Cases in Fluency Disorders, eds. Eggers K. Leahy M. (London, UK: Routledge, Taylor & Francis Group).

[ref50] Kösters M. P. Chinapaw M. J. Zwaanswijk M. van der Wal M. F. Koot H. M. (2015). Structure, reliability, and validity of the revised child anxiety and depression scale (RCADS) in a multi-ethnic urban sample of Dutch children. BMC Psychiatry 15:132. doi: 10.1186/s12888-015-0509-7, 26100511 PMC4477605

[ref51] Kraft S. J. Lowther E. Beilby J. (2019). The role of effortful control in stuttering severity in children: replication study. Am. J. Speech Lang. Pathol. 28, 14–28. doi: 10.1044/2018_AJSLP-17-0097, 30517950 PMC6503866

[ref52] Kristal J. (2005). The Temperament Perspective: Working with Children's Behavioral Styles. Baltimore, MD, USA: Paul H. Brookes Publishing Co.

[ref53] Laurent J. Joiner T. E. Catanzaro S. J. (2011). Positive affect, negative affect, and physiological hyperarousal among referred and non-referred youths. Psychol. Assess. 23, 945–957. doi: 10.1037/a0024080, 21744972

[ref54] Levy Kardash O. Ohana H. Arden A. Benish-Weisman M. (2025). Relations between social anxiety and identity formation among adolescents: social participation and self-esteem as resilience resources. Youth Soc. 58, 336–368. doi: 10.1177/0044118X251363275

[ref55] Liu Q. Merrin G. J. Razza R. A. (2024). Reciprocal associations between maternal behaviors and children’s self-regulation during the transition from early to middle childhood. J. Child Fam. Stud. 33, 1602–1617. doi: 10.1007/s10826-023-02703-z

[ref56] Mairet K. Boag S. Warburton W. (2014). How important is temperament? The relationship between coping styles, early maladaptive schemas and social anxiety. Int. J. Psychol. Psychol. Ther. 14, 171–190.

[ref57] Manning W. Beck J. G. (2013). The role of psychological processes in estimates of stuttering severity. J. Fluen. Disord. 38, 356–367. doi: 10.1016/j.jfludis.2013.08.002, 24331243

[ref58] McAllister J. Kelman E. Millard S. (2015). Anxiety and cognitive bias in children and young people who stutter. Procedia. Soc. Behav. Sci. 193, 183–191. doi: 10.1016/j.sbspro.2015.03.258

[ref59] Mulcahy K. Hennessey N. Beilby J. Byrnes M. (2008). Social anxiety and the severity and typography of stuttering in adolescents. J. Fluen. Disord. 33, 306–319. doi: 10.1016/j.jfludis.2008.12.002, 19328982

[ref60] Onslow M. (2025). Stuttering and neurodiversity across the lifespan: a moveable feast. Am. J. Speech Lang. Pathol. 34, 3565–3570. doi: 10.1044/2025_AJSLP-25-00089, 41052100

[ref61] Palombo F. Del Gado F. Rugolo F. Lasaponara S. Busan P. Tomaiuoli D. . (2025). The role of anticipation and neuroticism in developmental stuttering. Front. Psychol. 16:1576681. doi: 10.3389/fpsyg.2025.1576681, 40470016 PMC12133894

[ref62] Parker J. G. Rubin K. H. Erath S. A. Wojslawowicz J. C. Buskirk A. A. (2015). “Peer relationships, child development, and adjustment: a developmental psychopathology perspective,” in Developmental Psychopathology, eds. Cicchetti D. Cohen D. J.. Hoboken, New Jersey: John Wiley & Sons, Inc.

[ref63] Riley G. D. (2009). Stuttering severity instrument: SSI-4. Pro-Ed: Austin.

[ref64] Rodgers N. H. Jackson E. S. (2021). Temperament is linked to avoidant responses to stuttering anticipation. J. Commun. Disord. 93:106139. doi: 10.1016/j.jcomdis.2021.106139, 34175560

[ref65] Rothbart M. K. (2011). Becoming who we are: Temperament and Personality in Development. New York: The Guilford Press.

[ref66] Rothbart M. K. Ahadi S. A. Hershey K. L. (1994). Temperament and social behavior in childhood. Merrill-Palmer Q. 40, 21–39.

[ref67] Rothbart M. K. Bates J. E. (2006). “Temperament,” in Handbook of Child Psychology: Social, Emotional, and Personality Development, eds. Eisenberg N. Damon W. Lerner R. M.. 6th ed (Hoboken, NJ, USA: John Wiley & Sons, Inc.), 99–166.

[ref68] Rothbart M. K. Derryberry D. (1981). Development of individual differences in temperament. Adv. Pers. Assess. 1, 37–86.

[ref69] Rothbart M. K. Ellis L. K. Rueda M. R. Posner M. I. (2003). Developing mechanisms of temperamental effortful control. J. Pers. 71, 1113–1144. doi: 10.1111/1467-6494.7106009, 14633060

[ref70] Rothbart M. K. Sheese B. E. Posner M. I. (2007). Executive attention and effortful control: linking temperament, brain networks, and genes. Child Dev. Perspect. 1, 2–7. doi: 10.1111/j.1750-8606.2007.00002.x

[ref71] Rubin D. B. (1987). Multiple Imputation for Nonresponse in Surveys. New York: John Wiley & Sons.

[ref72] Samson I. Lindström E. Sand A. Herlitz A. Schalling E. (2021). Larger reported impact of stuttering in teenage females, compared to males – a comparison of teenagers’ result on overall assessment of the speaker’s experience of stuttering (OASES). J. Fluen. Disord. 67:105822. doi: 10.1016/j.jfludis.2020.105822 33348210

[ref73] Samson I. Schalling E. Herlitz A. Lindström E. Sand A. (2022). A cross-sectional investigation of the impact of stuttering on Swedish females and males in childhood, adolescence, and young adulthood. J. Speech Lang. Hear. Res. 65, 4608–4622. doi: 10.1044/2022_jslhr-22-00043, 36399792

[ref74] Sanchis-Sanchis A. Grau M. D. Moliner A.-R. Morales-Murillo C. P. (2020). Effects of age and gender in emotion regulation of children and adolescents. Front. Psychol. 11:946. doi: 10.3389/fpsyg.2020.00946, 32528367 PMC7265134

[ref75] Sanmartín R. Inglés C. J. Vicent M. Gonzálvez C. Díaz-Herrero Á. García-Fernández J. M. (2018). Positive and negative affect as predictors of social functioning in Spanish children. PLoS One 13:e0201698. doi: 10.1371/journal.pone.0201698, 30071086 PMC6072041

[ref76] Smith A. Weber C. (2017). How stuttering develops: the multifactorial dynamic pathways theory. J. Speech Lang. Hear. Res. 60, 2483–2505. doi: 10.1044/2017_JSLHR-S-16-0343, 28837728 PMC5831617

[ref77] Somerville L. H. (2013). The teenage brain: sensitivity to social evaluation. Curr. Dir. Psychol. Sci. 22, 121–127. doi: 10.1177/0963721413476512, 24761055 PMC3992953

[ref78] Starkweather C. W. (2002). The epigenesis of stuttering. J. Fluen. Disord. 27, 269–288. doi: 10.1016/S0094-730X(02)00144-4, 12506446

[ref79] Starkweather C. W. Gottwald S. R. (1990). The demands and capacities model: II. Clinical applications. J. Fluen. Disord. 15, 143–157. doi: 10.1016/0094-730X(90)90015-K

[ref80] Taylor B. Jick H. Maclaughlin D. (2013). Prevalence and incidence rates of autism in the UK: time trend from 2004-2010 in children aged 8 years. BMJ Open 3:e003219. doi: 10.1136/bmjopen-2013-003219, 24131525 PMC3808754

[ref81] Theurel A. Gentaz E. (2018). The regulation of emotions in adolescents: age differences and emotion-specific patterns. PLoS One 13:e0195501. doi: 10.1371/journal.pone.0195501, 29879165 PMC5991707

[ref82] Thompson S. F. Zalewski M. Lengua L. J. (2014). Appraisal and coping styles account for the effects of temperament on pre-adolescent adjustment. Aust. J. Psychol. 66, 122–129. doi: 10.1111/ajpy.12048, 25821237 PMC4374447

[ref85] Tichenor S. E. Yaruss J. S. (2019). Group experiences and individual differences in stuttering. J. Speech Lang. Hear. Res. 62, 4335–4350. doi: 10.1044/2019_JSLHR-19-00138, 31830852

[ref83] Tichenor S. E. Gerwin K. L. Walsh B. (2023). Repetitive negative thinking in adolescents who stutter. J. Speech Lang. Hear. Res. 66, 3290–3306. doi: 10.1044/2023_JSLHR-23-00147, 37494925 PMC10558142

[ref84] Tichenor S. E. Walsh B. M. Gerwin K. L. Yaruss J. S. (2022). Emotional regulation and its influence on the experience of stuttering across the lifespan. J. Speech Lang. Hear. Res. 65, 2412–2430. doi: 10.1044/2022_JSLHR-21-00467, 35738025 PMC9584136

[ref86] Upadhyay P. Sharma K. Patil G. S. Thejesh R. (2025). Impact of social and attitudinal environment on participation experiences in life situations in adolescents and young adults with stuttering. Int. J. Med. Public Health 15, 415–418.

[ref87] van Buuren S. (2018). Flexible Imputation of Missing Data. 2nd Edn New York: Chapman and Hall/CRC.

[ref88] van Buuren S. Groothuis-Oudshoorn K. (2011). Mice: multivariate imputation by chained equations in R. J. Stat. Softw. 45, 1–67. doi: 10.18637/jss.v045.i03

[ref89] Vanryckeghem M. (2025). Stuttering and neurodiversity: a question of ableism or anti-ableism? One central premise: the person who stutters. J. Fluen. Disord. 87:106188. doi: 10.1016/j.jfludis.2025.106188 41406587

[ref90] von Elm E. Altman D. G. Egger M. Pocock S. J. Gøtzsche P. C. Vandenbroucke J. P. (2007). The strengthening the reporting of observational studies in epidemiology (STROBE) statement: guidelines for reporting observational studies. PLoS Med. 4:e296. doi: 10.1371/journal.pmed.0040296, 17941714 PMC2020495

[ref9006] Walden T. A. Frankel C. B. Buhr A. P. Johnson K. N. Conture E. G. Karrass J. M. (2012). Dual diathesis-stressor model of emotional and linguistic contributions to developmental stuttering. Journal of Abnormal Child Psychology, 40, 633–44. doi: 10.1007/s10802-011-9581-8, 22016200 PMC3740566

[ref91] Walsh B. M. Grobbel H. Christ S. L. Tichenor S. E. Gerwin K. L. (2023). Exploring the relationship between resilience and the adverse impact of stuttering in children. J. Speech Lang. Hear. Res. 66, 2278–2295. doi: 10.1044/2023_JSLHR-23-00012, 37390495 PMC10468119

[ref92] White I. R. Royston P. Wood A. M. (2011). Multiple imputation using chained equations: issues and guidance for practice. Statist. Med. 30, 377–399. doi: 10.1002/sim.4067, 21225900

[ref93] Winebrake D. A. Huth N. Gueron-Sela N. Propper C. Mills-Koonce R. Bedford R. . (2025). An examination of the relations between effortful control in early childhood and risk for later externalizing psychopathology: a bi-factor structural equation modeling approach. Child Psychiatry Hum. Dev. 56, 1190–1205. doi: 10.1007/s10578-024-01716-z, 38878149

[ref9004] World Health Organization. (2001). International classification of functioning, disability and health (ICF). World Health Organization.

[ref9005] Yairi E Ambrose N. (2013). Epidemiology of stuttering: 21st century advances. Journal of Fluency Disorders, 38, 66–87. doi: 10.1016/j.jfludis.2012.11.002 23773662 PMC3687212

[ref94] Yaruss J. S. Quesal R. W. (2008). Overall Assessment of the Speaker’s Experience of Stuttering (OASES). Minneapolis, MN: Pearson. 10.1016/j.jfludis.2006.02.002 16620945

[ref95] Yaruss J. S. Quesal R. W. (2016). Overall Assessment of the Speaker’s Experience of Stuttering. Westerville, OH, USA: Stuttering Therapy Resources, Inc.

